# Forecasting and Early Warning Systems for Dengue Outbreaks: Updated Narrative Review

**DOI:** 10.1590/0037-8682-0429-2025

**Published:** 2026-01-16

**Authors:** José Micael Ferreira da Costa, Alexandre Cunha Costa, Cleiton da Silva Silveira, Suellen Teixeira Nobre Gonçalves, Antonio Duarte Marcos, Luciano Pamplona de Góes Cavalcanti

**Affiliations:** 1 Universidade Federal do Ceará, Departamento de Engenharia Hidráulica e Ambiental, Fortaleza, CE, Brasil.; 2 Universidade da Integração Internacional da Lusofonia Afro-Brasileira, Instituto de Engenharia e Desenvolvimento Sustentável, Redenção, CE, Brasil.; 3 Universidade Federal do Ceará, Departamento de Saúde Comunitária, Fortaleza, CE, Brasil.

**Keywords:** Statistical models, Machine learning, Deep learning, Early aberration reporting system, Public health.

## Abstract

In this review, we examine dengue outbreak prediction and warning systems, highlighting their methodologies, variables, key findings, and existing gaps in the literature. The study was conducted in five stages: a literature survey, definition of thematic scope and eligibility criteria, exploratory review, systematization and categorization of findings, critical analysis, and comparative narrative synthesis. We selected 14 articles on prediction and seven on warning systems, encompassing statistical models, machine learning, and deep learning, as well as systems applied in various countries, with a particular focus on Brazil. The results indicated that meteorological and climatic variables are the most frequently used, followed by epidemiological and entomological data. Models such as Random Forest and Long Short-Term Memory demonstrated superior predictive performance, especially for short-term forecasts of up to 1 week. Among the warning systems, classical methods, such as the Early Aberration Reporting System, offer simplicity and speed but provide shorter lead times. In contrast, systems such as EWARS-TDR and ADSEWS excel by integrating multiple data sources and providing longer lead times (up to 13 weeks). Despite considerable advancements, challenges related to data quality and availability, model replicability across different contexts, and implementation persist in public health systems.

## INTRODUCTION

Dengue is a viral disease transmitted by mosquitoes of the *Aedes* genus, primarily *Aedes aegypti*. With four circulating viral serotypes (DENV-1 to DENV-4), it represents a major public health challenge in tropical and subtropical regions worldwide^1^. Billions of people are estimated to live in at-risk areas, with 50-100 million new infections reported annually in over 80 countries, particularly those with hot and humid climates. These countries often exhibit environmental and socioeconomic conditions conducive to vector proliferation and recurrent seasonal outbreaks, which frequently strain their healthcare systems^2^ ^-^ ^8^.

In this context, prediction and warning systems for dengue outbreaks are essential tools for developing prevention and control strategies. They enable more timely interventions by health authorities, helping reduce disease-related mortality. Various methodological approaches have been employed to forecast the incidence of dengue, ranging from traditional statistical techniques to advanced machine learning (ML) and deep learning (DL) models. These models are based on heterogeneous data, including climatic, epidemiological, entomological, demographic, and socioeconomic factors^9^ ^-^ ^18^.

Dengue outbreak prediction systems are designed to estimate the probability, magnitude, and timing of future outbreaks, allowing for proactive planning of prevention and control measures before a substantial rise in cases occurs^19^. In contrast, outbreak warning systems operate reactively, detecting signals and anomalies that indicate an outbreak is imminent or already underway^20^. Together, predictions and warnings form a complementary strategy: predictions guide long-term planning, whereas warnings trigger rapid response actions^21^ ^,^ ^22^.

Although previous systematic reviews^23^ ^-^ ^25^, have synthesized advances in dengue forecasting models, this review extends this body of work by integrating the analysis of predictive algorithms with their operational applicability in early warning systems. This approach highlights the connection between statistical performance and real-world usability, providing a more comprehensive understanding of how forecasting tools can support public health decision-making and epidemic preparedness.

This study aimed to conduct an exploratory analysis of recent literature on dengue outbreak prediction and warning systems. It sought to identify and quantify the primary techniques, models, and indices adopted in the analyzed studies, refine the available information, highlight existing research gaps, and identify advancements in the development and application of these systems across different endemic regions. 

## METHODS

This narrative review with systematized elements^26^ was designed to analyze and compare the strategies, predictive models, and warning systems used for forecasting dengue outbreaks. The methodological process was structured into five interrelated but distinct stages to ensure clarity and transparency in the workflow: 1) literature survey: identification of all potential sources; 2) definition of thematic scope and eligibility criteria; 3) exploratory review: full-text reading and extraction of relevant data; 4) systematization and categorization of findings (including model type, data used, performance, and country); and 5) critical analysis and comparative narrative synthesis.

Initially, a comprehensive literature survey was conducted across major scientific databases, including PubMed, Scopus, Web of Science, SciELO, and Google Scholar, complemented by grey literature, institutional reports, and digital media sources. The search strategy combined controlled vocabulary and free-text terms using Boolean operators, as follows: (“dengue” AND (“forecast*” OR “predict*” OR “early warning system*” OR “alert system”)). Searches were applied to titles, abstracts, and descriptors (Medical Subject Heading terms) when available. To capture the most recent methodological developments in dengue forecasting, the search was limited to peer-reviewed publications from 2021 to 2025, written in English, Portuguese, or Spanish. The last search update was conducted on August 13, 2025.

Following the search, the thematic scope and eligibility criteria were defined to include studies that described, applied, or evaluated predictive or early warning systems for dengue outbreaks, particularly those integrating epidemiological, climatic, entomological, socioeconomic, or environmental variables. Only peer-reviewed articles were considered for inclusion. The exclusion criteria included studies not directly related to dengue, non-predictive or descriptive-only studies, and studies without accessible full text. During the review process, studies published in potentially predatory journals-identified through recognized listings and journal assessments (https://www.predatoryjournals.org/the-list/journals)-were excluded from the final set of included articles. Additional references were identified through manual screening of the bibliographies of the selected papers to ensure comprehensive coverage of relevant sources.

**FIGURE 1:**
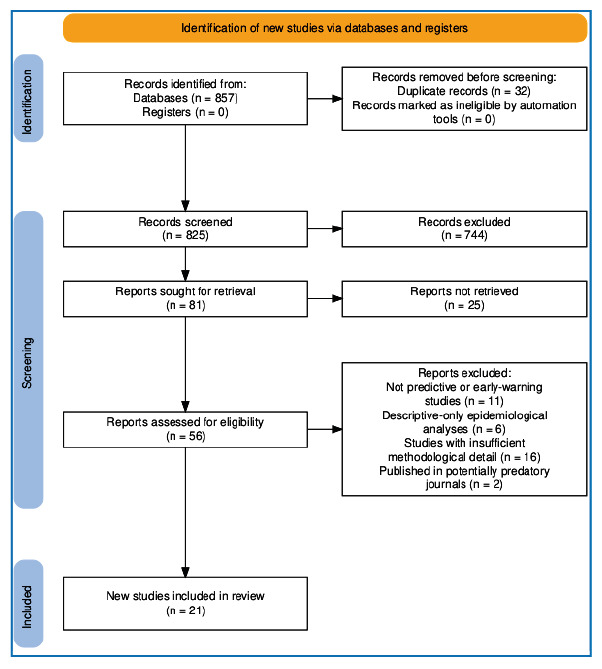
PRISMA-adapted flow diagram illustrating the identification, screening, eligibility, and inclusion stages of the reviewed studies. Generated using the PRISMA 2020 Flow Diagram Generator^27^ (https://estech.shinyapps.io/prisma_flowdiagram/)

To enhance methodological transparency, a simplified PRISMA-adapted^27^ flow diagram was prepared to illustrate the stages of identification, screening, eligibility assessment, and final inclusion of both forecasting and early warning system studies (Figure 1).

Subsequently, an exploratory review involving full-text reading and systematic extraction of relevant information was conducted. For each included study, data were collected on the objectives, methodological design, modeling approach, data inputs, and reported performance metrics. The extracted data were then systematized and categorized based on predefined analytical dimensions specifically applied to the selection and comparison of dengue forecasting models: (1) model type (statistical, ML, or DL); (2) variables used (climatic, entomological, epidemiological, socioeconomic, and environmental); (3) performance indicators (accuracy, sensitivity, lead time, or type of validation); (4) geographical scope (country and spatial scale); and (5) publication year.

However, these classification criteria were not consistently applied to dengue early warning systems. This is because such studies generally focused on the operational implementation of existing forecasting models rather than the development or validation of predictive algorithms, which justifies the absence of methodological parameters in this category. Data organization and coding were performed using Excel spreadsheets and reference management tools, such as Zotero and Mendeley, enabling descriptive and comparative synthesis. Frequency counts and qualitative assessments were applied to identify patterns in modeling strategies, variables, and validation procedures across studies.

Finally, a critical and comparative narrative synthesis was developed to summarize the main findings, highlight methodological tendencies, and discuss current limitations and research gaps. Although no quantitative synthesis (such as meta-analysis) was performed, this approach ensured a rigorous and transparent process, consistent with a narrative review incorporating systematic elements.

## RESULTS

We initially identified 21 articles, comprising 14 studies that focused on prediction and seven on outbreak warning systems. Among these, four were reviews-three quantitative^23^ ^-^ ^25^ and one qualitative^20^.

Accordingly, the results section is categorized into two subsections, one for each system type (prediction and warning), with both systems integrated in the final considerations section.

### Dengue outbreak prediction models

Dengue outbreak prediction systems have been established as strategic tools for public health, enabling authorities to anticipate incidence peaks and implement preventive measures more effectively. These systems employ various analytical approaches^28^ ^,^ ^29^, ranging from traditional statistical models (SMs) to advanced techniques, such as ML, DL, and artificial intelligence (AI) (Table 1). They utilize historical and real-time data to identify future trends and risks, supporting strategic planning and offering prediction time horizons that may range from weeks to months, and may even extend to years^28^.

**TABLE 1:** Main types of techniques and models/algorithms used for dengue outbreak prediction

Main category	Subtype / Technique	Examples of Models / Algorithms
Statistical Models (SM)	Regression	Linear, Logistic, Poisson, Negative Binomial, Least Absolute Shrinkage and Selection Operator (LASSO)
	Additive Models	Generalized Additive Model (GAM), Generalized Linear Model (GLM)
	Time Series Regression	AutoRegressive Integrated Moving Average (ARIMA), Seasonal ARIMA (SARIMA), Seasonal ARIMA with eXogenous variables (SARIMAX), Vector AutoRegression (VAR), Prophet, Distributed Lag Non-Linear Model (DLNM)
Machine Learning (ML)	Tree-Based Classifiers	Decision Tree (DT), Random Forest (RF), Extreme Gradient Boosting (XGBoost), Adaptive Boosting (AdaBoost), Gradient Boosting Machine (GBM), LightGBM, Boosted Regression Trees (BRT), Extra Trees Classifier, CatBoost Regressor
	Regression and Classification	Logistic Regression (LR), Support Vector Machine (SVM), Support Vector Regression (SVR), Principal Component Analysis - Support Vector Machine (PCA-SVM), k-Nearest Neighbors (kNN), Naïve Bayes, Bayes Network
	Artificial Neural Networks (ANN)	Feedforward Networks: Multi-Layer Perceptron (MLP)
Deep Learning (DL)	Deep Neural Networks	Deep Neural Network (DNN), Long Short-Term Memory (LSTM), Convolutional Neural Network (CNN), Recurrent Neural Network (RNN), Long Short-Term Memory with Attention (LSTM-ATT)
	CNN with Visual Data	CNN with Google Street View images
Ensemble Models	Combination of multiple models	RF, XGBoost, AdaBoost, GBM, BRT
Auxiliary Techniques	Dimensionality Reduction	Principal Component Analysis (PCA)
	Hybrid and Integrated Methods	ANN + ARIMA; PCA + SVM; CNN + LSTM; ARIMA + LSTM; etc.
Models with Exogenous Variables	Integration with External Factors	SARIMAX, GAM (climate, mobility), CNN (images), ML (climatic, socioeconomic, geographic), etc.

**Sources:** Joseph^37^ e Spiliotis^38^.

These systems primarily differ in terms of model complexity and the breadth of variables incorporated. Traditional SMs typically offer greater interpretability and require less computational power, making them well-suited for contexts with limited data availability. In contrast, ML and DL models excel at capturing nonlinear relationships and complex patterns, often achieving superior accuracy, especially when combined in hybrid or ensemble approaches. Therefore, the choice of an ideal system depends on the balance between precision, interpretability, data availability, and practical applicability in each local context^29^.

**FIGURE 2:**
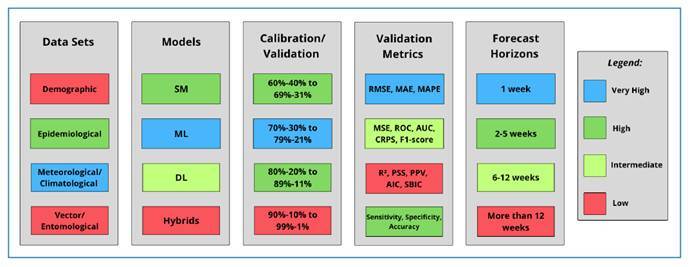
Quantification of the methodological characteristics identified in the 14 analyzed articles.

The three quantitative review articles^23^ ^-^ ^25^, along with 11 other original studies^4^ ^,^ ^6^ ^,^ ^30^ ^-^ ^38^, constituted the 14 studies evaluated. Their main findings are presented in Table 2, which highlights predictor variables, model types, ratios between calibration and validation periods, model validation metrics, and prediction time horizons. An extended version, containing main trends and complete descriptive details, is provided in Supplementary Material Table S1. A summary and quantification of these results are illustrated in Figure 2.

**TABLE 2:** Quantitative summary of datasets, models, calibration and validation periods, validation metrics, and prediction horizons across 14 dengue-forecasting studies. Numbers (in parentheses) and percentages (in brackets) represent the study frequency (number of articles) and relative contribution (%), respectively.

Variables used	Models analyzed	Calibration/Validation period	Validation metrics	Prediction horizon
Demographic: (3) [21,43%]	DL: (5) [35,71%]	60‒40% to 69‒31%: (3) [21,43%]	Accuracy: (3) [21,43%]	1 week: (7) [50,00%]
Epidemiological: (10) [71,43%]	ML: (9) [64,29%]	70‒30% to 79‒21%: (4) [28,57%]	AIC: (1) [7,14%]	2‒5 weeks: (6) [42,86%]
			AUC: (3) [21,43%]	
Meteorological/Climatological: (12) [85,71%]	SM: (7) [50,00%]	80‒20% to 89‒11%: (3) [21,43%]	CRPS: (3) [21,43%]	6‒12 weeks: (4) [28,57%]
			F1-score: (3) [21,43%]	
Vector/Entomological: (3) [21,43%]	Hybrids: (3) [21,43%]	90‒10% to 99‒1%: (1) [7,14%]	MAE: (6) [42,86%]	> 12 weeks: (1) [7,14%]
			MAPE: (6) [42,86%]	
			MSE: (2) [14,29%]	
			PPV: (1) [7,14%]	
			PSS: (1) [7,14%]	
			R²: (1) [7,14%]	
			RMSE: (6) [42,86%]	
			ROC: (3) [21,43%]	
			SBIC: (1) [7,14%]	
			Sensitivity: (4) [28,57%]	
			Specificity: (4) [28,57%]	

**SUPPLEMENTARY MATERIAL TABLE S1:** Descriptive characteristics of studies using different algorithms for dengue prediction. Numbers in parentheses indicate the quantification of articles.

Author	Country (Geographical Scope)	Study design (No. of articles analyzed)	Variables used	Models analyzed	Best-performing models	Calibration/Validation period	Validation metrics	Prediction horizon
Baharom et al.^23^	Malasya (Global)	Review (17)	Meteorological/Climatological; Entomological; Epidemiological; Demographic.	ML (5) SM (4) SM & ML (4)	Bayes Network; SVM; SVR.	6‒10 years (9); Up to 5 years (6); 11‒20 years (2).	Sensitivity (8); Specificity (4); Positive predictive value (PPV) (3);MAE (4);MAPE (2); MSE, PSS, CRPS, R² (1).	1‒12 weeks (5); 1‒3 months (2); 2‒5 weeks (1); 4 weeks (1).
da Silva Neto et al.^24^	Brazil (Global)	Review (15)	N / A	ML (12); DL (1): 13 total	DT, RF, AdaBoost; ANN; SVM.	N / A	Sensitivity, Accuracy, Specificity, Precision;ROC, AUC;F1-Score.	N / A
Leung et al.^25^	Australia(Global)	Review (64)	Meteorological/Climatological; Climate change; Entomological; Demographic.	SM (41); ML (17); SM & ML (6); Total models (99)	N / A	N / A	RMSE; MSE;MAPE;ROC.	N / A
Roster el at^30^	Brazil (Brazil)	Original Article	Epidemiological; Meteorological/Climatological.	ML: RF; GBM (Regressor); SVR; ANN.	RF	2007‒2015 (9 years); 2016‒2019 (4 years).	RMSE; MAE	4 weeks
Koplewitz et al^31^	EUA (Brazil)	Original Article	Epidemiological; Google search frequencies for dengue-related queries; Meteorological/Climatological.	ML: RF; SM: LASSO	RF	01/2011‒06/2012 (1.5 years).	RMSE	1, 3, 6, and 8 weeks
Sanchez-Gendriz et al^32^	Brazil (Brazil: Natal-RN)	Original Article	Entomological; Epidemiological.	DL: LSTM	LSTM (with Entomological data)	01/ 2016‒02/ 2019; 03/ 2019‒12/ 2019.	RMSE	1 week
Ong et al.^6^	Malaysia (Malaysia)	Original Article	Meteorological/Climatological; Entomological.	ML: XGBoost, AdaBoost, RF, SVM, Naïve Bayes, DT, and LR.	XGBoost, AdaBoost, RF.	70% (11 years, 7 months); 30% (4 years, 11 months).	ROC-AUC; Accuracy; F1-Score.	1 week
Karasinghe et al.^33^	Sri Lanka (Sri Lanka)	Original Article	Epidemiological	SM: ARIMA.	ARIMA (2,1,0) supplemented with an AR (16) term	01/2015‒08/2020 (5 years and 8 months); 09/2020‒12/2020 (4 months).	AIC, SBIC, and MAPE	1 week
Uduwanage et al.^4^	Sri Lanka (Sri Lanka)	Original Article	Meteorological/Climatological; Epidemiological.	DL: CNN; ML: XGBoost; SM: SARIMAX.	SARIMAX	01/2007‒04/ 2024.	RMSE	N / A
da Silva et al.^34^	Brazil/Germany (Brazil, Peru, & Colombia)	Original Article	Epidemiological; Meteorological/Climatological.	ML: RF	RF (with relative humidity)	08/2001‒12/2019; Best ratio (Brazil): 64‒80% (calibration); 20‒36% (validation).	MAE	1 week
Villela^35^	Brazil (Brazil)	Original Article	Epidemiological; Meteorological/Climatological.	SM: LASSO	LASSO (with Path Signatures)	2014‒2024; ⅔ (calibration); ⅓ (validation).	AUC; Sensitivity; Specificity.	Week 26‒50
Chen e Moraga^36^	Saudi Arabia (Brazil)	Original Article	Epidemiological; Meteorological/Climatological; Human mobility.	DL: LSTM-epidemiological; LSTM-climate; LSTM-climate + spatial (Human mobility).	LSTM- Climate + Spatial	2016‒2022 (7 years); 2023 (1 year).	MAE; MAPE; CRPS; Accuracy; Sensitivity; Specificity; F1 score.	4 weeks
Chen e Moraga^37^	Saudi Arabia (Brazil)	Original Article	Meteorological/Climatological; Spatial (lagged dengue cases from neighboring Brazilian states).	DL: LSTM (enhanced with SHAP).	LSTM-Climate+Spatial	2016‒2022 (7 years); 2023 (1 year)	MAE, MAPE, and CRPS	4‒12 weeks
Chen e Moraga^38^	Saudi Arabia (Brazil)	Original Article	Meteorological/Climatological; Epidemiological.	SM (6); ML (5); Ensemble (with best-performing methods)	ARIMA, SARIMAX; LSTM (short & medium term), Prophet (long term); LSTM + ARIMA.	2016‒2021 (6 years); 2022‒2023 (2 years).	MAE, MAPE, RMSE.	1, 2, 3, 4 (short), 8 (medium), and 12 (long) weeks

The key information collected from each of the 14 selected articles is summarized in Table 2 and Supplementary Material Table S1. These included three review articles, totaling 96 studies, in addition to 11 original research articles that were also evaluated. The main predictor variables used across these studies were categorized as meteorological/climatological, entomological, epidemiological, and demographic. Among these, meteorological and climatic variables were the most frequently used, followed by epidemiological, entomological, and demographic variables.

Regarding the models analyzed, SMs were prominent in one of the review articles^25^, whereas ML models predominated in the other reviews. Original articles also demonstrated a predominant use of ML models and a growing application of DL approaches. Among the representative models in these categories, Random Forest (RF) and Long Short-Term Memory (LSTM) were the most frequently cited for their superior performance, with five and four mentions, respectively. The most common data split ratios for calibration/validation were 70‒30% and 79‒21%. The most widely used validation metrics were Root Mean Squared Error (RMSE), Mean Absolute Error (MAE), and Mean Absolute Percentage Error. Finally, the most common prediction time horizon was 1 week. 

Figure 2 illustrates the methodological criteria from the analyzed articles, as detailed in Table 2. This figure synthesizes the results to highlight the frequency of use for each item, categorized as very high, high, intermediate, or low. Notably, the counts for these items are not mutually exclusive, as a single study could be tallied under multiple categories.

### Early warning systems

Early warning systems often rely on pre-established thresholds for critical variables, such as sudden increases in case notifications, rise in entomological indices, or abrupt changes in climatic conditions^20^. When these thresholds are exceeded, an alert is issued to health authorities, who can then trigger rapid response actions, including intensified insecticide fogging, community mobilization campaigns, and inspection of breeding sites. The most commonly used warning systems in the recent literature are summarized in Table 3 ^39^ ^,^ ^40^. The seven selected studies, which focused on outbreak warning systems, consisted of six original articles^5^ ^,^ ^21^ ^,^ ^22^ ^,^ ^41^ ^-^ ^43^ and one review^20^. 

**TABLE 3:** Most employed dengue outbreak warning systems.

System	Creator/origin	Scope/application	Alert type/approach	Typical lead time	Key features
Early Aberration Reporting System (EARS)	CDC (USA)	General epidemiological surveillance	SM for anomaly detection in time series	1-2 weeks (operational)	Provides rapid, simple alerts based solely on epidemiological data; detects recent anomalies rather than predicting future outbreaks.
Early Warning, Alert, and Response System (EWARS)	WHO	Humanitarian emergencies (disasters, crises)	SM for immediate detection and response	1-6 weeks (predictive)	Features multi-pathogen surveillance; integrates meteorological and epidemiological data.
Special Programme for Research and Training in Tropical Diseases (EWARS-TDR)	TDR/WHO	Endemic arboviruses (dengue, Zika, chikungunya)	Regression-based SM with multiple alarm signals (climatic, entomological, epidemiological)	1-13 weeks (predictive)	An optimized version of EWARS allows for the planning of interventions.
Advanced Dengue Surveillance and Early Warning System (ADSEWS)	Recent researchers/Endemic countries	Advanced endemic dengue surveillance	SM + ML applied to epidemiological, climatological time series, etc. May feature a binary alert (outbreak / no outbreak).	1-13 weeks (predictive, with greater robustness)	An evolution of EWARS-TDR; integrates ML and multiple data sources, providing more accurate predictions and a longer lead time for control actions.

**Sources:** WHO^39^ e WHO & TDR^40^.

A study^41^ evaluated the implementation of the Early Aberration Reporting System (EARS) as an early warning system for dengue outbreaks in Brazil through anomaly detection, using primary healthcare data on consultations for acute febrile syndromes. Three variations of the algorithm (C1, C2, and C3) were tested, and the observed values were compared with the mean and standard deviation of previous days to identify anomalies. EARS-C3 showed the best performance, balancing sensitivity and specificity by accumulating deviations over 3 consecutive days and reducing false positives. Consequently, the system successfully detected outbreaks up to 4 weeks earlier than official notifications, offering a simple statistical tool to support epidemiological surveillance and enable faster responses in dengue control.

Another study^42^ evaluated the operational implementation of the Early Warning, Alert, and Response System-Special Program for Research and Training in Tropical Diseases (EWARS-TDR) for dengue outbreaks in Mexico, which has been incorporated into the national surveillance system since 2014. The system applies distributed-lag nonlinear models combined with the Bayesian INLA framework, integrating epidemiological, entomological, and climatic indicators to calculate weekly outbreak probabilities up to 12 weeks in advance, triggering alerts when epidemic thresholds are exceeded. The results indicate that EWARS-TDR strengthened coordination between epidemiological surveillance, vector control, and climate institutions, promoting faster and more robust responses.

Another study assessed^43^ the performance of EWARS-TDR in predicting dengue, chikungunya, and Zika outbreaks in districts of Mexico and Colombia, using epidemiological, climatic, and entomological data from national surveillance systems. The methodology involved defining calibration and validation periods to measure sensitivity, positive predictive value (PPV), and lead time between alarm signals and outbreak occurrence. The results showed satisfactory performance for dengue (sensitivity up to 100% and PPV of 83% in Mexico, and 92% and 68% in Colombia, respectively). The system also showed good predictive performance for chikungunya (93% sensitivity and 92% PPV) and Zika (sensitivity near 100%, with PPV varying between 54% and 100%). EWARS-TDR demonstrated the ability to provide a 3-13-week lead time for implementing control measures, reinforcing its potential as an early warning tool, although performance varied depending on the epidemiological context and specific disease.

In another stydy^22^, SM and ML were used to develop an Advanced Dengue Surveillance and Early Warning System (ADSEWS) to predict dengue outbreaks 1 week in advance. The system used a time series of epidemiological and climatological data, including precipitation measured by a network of rain gauges strategically distributed at 3-4 km intervals in critical areas. Among the tested models, RF demonstrated the best performance, achieving 95% accuracy when epidemiological, entomological, and climatological data were included, and maintaining 92% accuracy even without entomological data, which are more complex and time-consuming to collect. The viability of the system was enhanced by integrating Internet of Things technologies, which are networked devices capable of collecting and transmitting data in real time. The climatological data obtained in this manner, combined with epidemiological records, were processed by the predictive model (RF), enabling the automatic issuance of alerts when conditions similar to those preceding previous outbreaks were detected.

One study^21^ evaluated the ADSEWS in Ningbo, China, in 2023, highlighting its capacity for rapid detection and response to outbreaks, particularly imported cases. The methodology consisted of an operational performance analysis comparing the new system with the traditional notification model, based on the collection of clinical and hematological data from suspected patients and official notification records. This approach allowed for an assessment of the time elapsed between initial detection and communication to health authorities. Although the traditional system relied heavily on formal medical notifications and experienced longer information-sharing delays, the advanced system incorporated multiple real-time data sources, reduced response times, increased sensitivity for imported cases, and improved integration between healthcare units and epidemiological surveillance.

Ningrum et al.^5^ developed an AI-based ADSEWS using a spatiotemporal approach, integrating weekly epidemiological data with meteorological and climatological variables from districts in Semarang, Indonesia. The model performed both binary outbreak prediction (outbreak/no outbreak), providing early classification at the district level 1 week in advance, and continuous incident case prediction, estimating the expected number of cases for the same interval. The results showed that the Extra Trees Classifier performed best for outbreak prediction (accuracy = 89.25%; AUROC = 95.29%), whereas the spatiotemporal model was more effective for incident case prediction (R^2^ = 0.5621; RMSE = 1.0891).

Finally, a systematic review^20^ of 30 articles evaluated the quantitative models applied in Africa for predicting dengue and *Aedes* abundance, highlighting their potential and limitations for developing early warning systems. Most studies have focused on traditional SMs based on entomological and environmental data, with limited use of human case data and scarce application of advanced techniques, such as ML models. A lack of real-time data integration and robust model validation was also observed, compromising their practical applicability. The authors concluded that, despite some progress, none of the reviewed studies had developed an explicit early warning system for dengue, underscoring the need for more integrated, modern, and validated approaches to support epidemiological surveillance on the continent.

## FINAL CONSIDERATIONS

The analysis of the reviewed studies highlights considerable advances in dengue outbreak prediction and warning systems, driven by the adoption of ML and DL techniques. Traditional SMs, such as linear regressions and ARIMA, remain relevant in contexts with limited data availability owing to their simplicity and interpretability, as demonstrated previously^10^. Nonetheless, more complex models-such as RF and LSTM-stand out for their superior accuracy and ability to capture non-linear patterns and complex interactions between climatic, epidemiological, and socioeconomic variables. This shift illustrates an ongoing methodological transition, where emphasis is moving from statistical explanation toward predictive robustness, albeit at the expense of higher computational demands and reduced interpretability.

Our findings corroborate a trend reported in Bangladesh^44^, where RF, XGBoost, and LightGBM algorithms were evaluated within an early warning framework. LightGBM, particularly when combined with SHAP values, demonstrated the best balance between predictive accuracy and interpretability-an essential attribute for public health applications. The study further identified critical thresholds for temperature (minimum ~22-25 °C, maximum ~32-34 °C), relative humidity (75-85%), precipitation (~10 mm), and wind speed (~12 m/s) associated with heightened dengue risk, in addition to land use and population density. These results reinforce the central role of environmental and social variables, aligning with our study, which found that meteorological and climatic predictors are most frequently used, emphasizing the added value of epidemiological and entomological data.

In this context, hybrid and ensemble approaches have emerged as key differentiators that integrate multiple models to improve predictive robustness. The One Health framework underscores the importance of incorporating human, environmental, and vector dimensions, arguing that the complexity of dengue requires models capable of addressing multiple layers of risk. Nevertheless, persistent limitations such as restricted data availability and limited model replicability across diverse contexts highlight the need for locally tailored predictive systems. No single model has proven universally superior; rather, the most promising strategy is context-specific adaptation, which leverages available data and combines complementary algorithms.

However, a critical appraisal of these studies reveals methodological heterogeneity, particularly regarding validation and control of overfitting. Few studies have employed external validation or cross-regional testing, raising concerns about the generalizability of the model. Moreover, several ML and DL models were trained on relatively small datasets, increasing overfitting risks and limiting robustness under real-world conditions. Interpretability remains a persistent challenge, particularly for DL architectures, where complex parameterization hinders transparency and reproducibility.

This framework allowed us to examine how different combinations of predictors and algorithms affect the real-world performance of dengue prediction systems^45^ ^-^ ^47^, as expressed through metrics such as accuracy, sensitivity, and lead-time reliability. Studies exclusively using climatic predictors (including temperature, rainfall, and humidity) typically performed well for short-term forecasts when employing ensemble tree-based models (such as RF), whereas those integrating epidemiological and entomological data achieved greater robustness and adaptability for medium-term horizons through deep learning architectures, such as LSTM. In contrast, purely SMs (such as ARIMA) showed limited performance beyond 1 month, highlighting the trade-offs between model complexity, data requirements, and operational applicability. 

Beyond predictive performance, operational implementation faces technical barriers that have rarely been addressed in the reviewed studies. Real-time data latency, uncertainty quantification, and integration of heterogeneous data sources remain major obstacles to building responsive and reliable early warning systems. In many cases, governance issues, data-sharing restrictions, and insufficient computational infrastructure impede the transition from research prototypes to fully functional operational tools. This comparative perspective underscores the need for integrated, data-diverse models^48^ that not only enhance forecasting reliability and decision-making in dengue early warning systems but also foster closer collaboration between data scientists, epidemiologists, and public health authorities. Strengthening these partnerships is essential to ensure the scalability, operational integration, and long-term sustainability of predictive systems in real-world public health infrastructures.

Regarding warning systems, this review shows that classical methods such as the EARS remain valuable owing to their simplicity and rapid anomaly detection, although they operate reactively with short lead times. More advanced systems, such as EWARS-TDR and ADSEWS, demonstrate greater potential by integrating multiple variables and ML techniques, enabling alerts with longer lead times-up to 13 weeks in some cases. Experiences from Mexico, Colombia, Brazil, and China illustrate that these systems can accelerate responses by incorporating epidemiological, climatic, and entomological data in near real time. However, widespread implementation faces barriers, including dependence on complete datasets, lack of methodological standardization, and limited technological infrastructure within public health systems.

In Brazil, dengue continues to pose a major public health challenge, with recurrent epidemic cycles that strain health services. To address this, the Ministry of Health, in partnership with research institutions, has expanded its predictive and early warning initiatives. A prominent example is InfoDengue^49^, which integrates epidemiological data from SINAN, real-time notifications, meteorological indicators (such as rainfall and temperature), and internet search trends to produce weekly transmission risk maps. 

In operational terms, the forecasts generated by InfoDengue are directly linked to decision-making processes within municipal and state health departments. When the system detects an increased probability of transmission, health authorities can intensify vector-control measures-such as targeted larvicide application, inspection of breeding sites, mobilization of community agents, and allocation of additional personnel and supplies to high-risk areas. This integration of predictive analytics with routine surveillance helps optimize resources and enhances the timeliness and geographic precision of outbreak responses. Several states and municipalities have also implemented predictive tools, confirming their practical applicability^32^ ^,^ ^41^ ^,^ ^50^ ^-^ ^54^.

Dengue remains one of the most pressing public health challenges in Brazil. The country recorded its largest dengue epidemic in 2024, with over six million probable cases and 4,000 deaths^55^ ^-^ ^56^. Nonetheless, challenges persist, including uneven coverage across the country, underreporting of cases, delays in SINAN data entry, and limited integration of forecasts into local decision-making. In many municipalities, predictive outputs are still used reactively rather than proactively because of insufficient technical capacity and fragmented communication between epidemiological surveillance units and vector-control teams. 

Although Brazil has robust warning tools, the priority now lies in consolidating their operational use-ensuring that alerts systematically trigger pre-defined intervention protocols, facilitate intersectoral coordination, and guide the allocation of human and material resources in advance of epidemic peaks. A study^3^ conducted in Brazilian capital cities found that greater outpatient capacity was associated with lower dengue mortality, underscoring the essential role of primary and secondary care in mitigating outbreaks.

Another important challenge relates to differential diagnosis. A study^57^ highlighted the frequent clinical overlap between dengue and a wide range of infectious (including malaria, leptospirosis, chikungunya, Zika, influenza, and COVID-19) and non-infectious (such as rheumatological, hematological, and gastrointestinal) conditions. Non-specific manifestations, such as fever, rash, and thrombocytopenia, often hinder timely differentiation, leading to diagnostic errors that delay treatment and outbreak alerts. These findings underscore that even the most accurate predictive systems will only be effective if integrated into healthcare networks capable of transforming information into timely interventions.

Despite including studies from multiple regions, this review is geographically biased toward research conducted in Asia and Latin America, particularly Brazil, where dengue surveillance and modeling are more extensively developed. This regional predominance reflects the high frequency and intensity of dengue outbreaks in these areas, which has driven greater research interest and data availability. In contrast, studies from Africa and the Pacific remain underrepresented, largely because of their limited surveillance capacity and fewer indexed publications. This imbalance may restrict the global generalizability of our findings and underscores the need for expanded predictive research and development of early warning systems in these regions^58^.

Collectively, these methodological limitations highlight the need for standardized validation frameworks and transparent reporting of model performance, including uncertainty estimates. Despite technical progress, interpretability remains a central concern, as many advanced ML and DL models operate as “black boxes,” creating distrust among health professionals and decision makers. Explainable AI techniques, such as SHAP^59^ ^,^ ^60^ and LIME^60^ ^,^ ^61^, along with Geographic Information Systems used for dengue surveillance^62^, offer promising pathways to reconcile predictive accuracy with interpretability. Furthermore, the lack of consistent model validation across diverse geographic and epidemiological settings limits scalability and generalizability. Addressing these challenges requires stronger methodological standardization, open-data protocols, and real-time adaptability to enhance the replicability and operational reliability of predictive systems^63^.

## CONCLUSION

This review synthesizes recent advances in dengue outbreak prediction and warning systems, focusing on methodological approaches, predictor variables, and emerging innovations. Although traditional SMs, such as regression and ARIMA, remain useful in contexts with limited data, ML and DL techniques are increasingly prominent. Among these, RF and LSTM models stand out for their ability to capture non-linear patterns and complex interactions, particularly when integrating meteorological, epidemiological, entomological, and geographic variables to enhance predictive accuracy. More advanced warning systems, such as EWARS-TDR and ADSEWS, also demonstrate superior precision and longer lead times than classical approaches, such as EARS, thereby expanding opportunities for rapid and effective dengue response.

Despite these advances, key challenges persist, including reliance on complete, high-quality databases, variability in model performance across regions, and difficulties in implementing complex approaches in resource-limited public health systems. Future studies should prioritize the continued development of ML- and DL-based models, coupled with adaptive warning systems, such as ADSEWS, while incorporating more diverse and representative predictor variables. A persistent gap remains between research-oriented predictive prototypes and their incorporation into routine epidemiological surveillance and decision-making pathways. 

Equally critical is the effective integration of these predictive tools into healthcare networks, which requires interoperability with existing information systems, standardized data-sharing protocols, and trained personnel capable of translating forecasts into targeted vector-control and resource-allocation actions. These steps are essential to ensure that forecasts and alerts not only anticipate outbreaks but also inform timely, actionable interventions to strengthen dengue control.

## Data Availability

Data-in-article: Research data is available in the body of the document (whole manuscript, and more specifically in Table 2 and Figure 2).
